# AREA DEPRIVATION INDEX EFFECTS ON LONGITUDINAL REPORTS OF PHYSICAL HEALTH AMONG OLDER BLACK ADULTS

**DOI:** 10.1093/geroni/igac059.455

**Published:** 2022-12-20

**Authors:** Alexa Allan, Alyssa Gamaldo, Regina Wright, Adrienne Aiken Morgan, Anna Lee, Jason Allaire, Roland J Thorpe, Jr., Keith Whitfield

**Affiliations:** The Pennsylvania State University, University Park, Pennsylvania, United States; Pennsylvania State University, State College, Pennsylvania, United States; University of Delaware, Newark, Delaware, United States; University of North Carolina at Chapel Hill, Chapel Hill, North Carolina, United States; North Carolina A&T State University, Greensboro, North Carolina, United States; North Carolina State University, Raleigh, North Carolina, United States; Johns Hopkins University, Baltimore, Maryland, United States; University of Nevada, Las Vegas, Las Vegas, Nevada, United States

## Abstract

Despite the association of neighborhood quality with poorer adult health, limited research has explored the association between neighborhood disadvantage, e.g., Area Deprivation Index (ADI), and older Black adults’ health, prospectively. The Baltimore Study of Black Aging (n = 424) was utilized to examine the association between ADI and prospective physical health. Multiple regression analyses, covarying for age, sex, education, and income, showed that living in a neighborhood with greater disadvantage was significantly associated with decreasing average heart rate from wave 1 to wave 2 (approximately 33 months apart) (p < .05). This result was specifically significant for women living in neighborhoods with greater disadvantage. Additionally, findings indicated that living in a neighborhood with greater disadvantage was significantly associated with increased difficulty with activities of daily living (p < .01). More research is needed to examine the implications of neighborhood context on older Black adults’ health prospectively.

